# Cellular and Molecular Bases of the Initiation of Fever

**DOI:** 10.1371/journal.pbio.0040284

**Published:** 2006-08-22

**Authors:** Alexandre A Steiner, Andrei I Ivanov, Jordi Serrats, Hiroshi Hosokawa, Allison N Phayre, Jared R Robbins, Jennifer L Roberts, Shigeo Kobayashi, Kiyoshi Matsumura, Paul E Sawchenko, Andrej A Romanovsky

**Affiliations:** 1 Systemic Inflammation Laboratory, Trauma Research, St. Joseph's Hospital and Medical Center, Phoenix, Arizona, United States of America; 2 Department of Pathology and Laboratory Medicine, Emory University, Atlanta, Georgia, United States of America; 3 Laboratory of Neuronal Structure and Function, The Salk Institute for Biological Studies, La Jolla, California, United States of America; 4 Department of Intelligence Science and Technology, Graduate School of Informatics, Kyoto University, Kyoto, Japan; 5 Osaka Institute of Technology, Hirakata-City, Osaka, Japan; Beth Israel Hospital, United States of America

## Abstract

All phases of lipopolysaccharide (LPS)-induced fever are mediated by prostaglandin (PG) E_2_. It is known that the second febrile phase (which starts at ~1.5 h post-LPS) and subsequent phases are mediated by PGE_2_ that originated in endotheliocytes and perivascular cells of the brain. However, the location and phenotypes of the cells that produce PGE_2_ triggering the first febrile phase (which starts at ~0.5 h) remain unknown. By studying PGE_2_ synthesis at the enzymatic level, we found that it was activated in the lung and liver, but not in the brain, at the onset of the first phase of LPS fever in rats. This activation involved phosphorylation of cytosolic phospholipase A_2_ (cPLA_2_) and transcriptional up-regulation of cyclooxygenase (COX)-2. The number of cells displaying COX-2 immunoreactivity surged in the lung and liver (but not in the brain) at the onset of fever, and the majority of these cells were identified as macrophages. When PGE_2_ synthesis in the periphery was activated, the concentration of PGE_2_ increased both in the venous blood (which collects PGE_2_ from tissues) and arterial blood (which delivers PGE_2_ to the brain). Most importantly, neutralization of circulating PGE_2_ with an anti-PGE_2_ antibody both delayed and attenuated LPS fever. It is concluded that fever is initiated by circulating PGE_2_ synthesized by macrophages of the LPS-processing organs (lung and liver) via phosphorylation of cPLA_2_ and transcriptional up-regulation of COX-2. Whether PGE_2_ produced at the level of the blood–brain barrier also contributes to the development of the first phase remains to be clarified.

## Abstract 2

The authors show that peripherally-produced COX2 plays an important role in the earliest stages of fever.

## Introduction

Fever is an ancient host-defense response and a common symptom of infection and systemic inflammation. Since Milton and Wendlandt [1] discovered the pyrogenic activity of prostaglandins (PGs) of the E series, and Vane [2] found that nonsteroidal anti-inflammatory drugs block fever by inhibiting PG synthesis, it has been accepted that fever is mediated by PGs, specifically PGE_2_ [3–6]. PGE_2_ synthesis occurs in three steps: (1) membrane phospholipids are converted to arachidonic acid by phospholipase A_2_ (PLA_2_); (2) arachidonic acid is converted to PGH_2_ by cyclooxygenase (COX); and (3) PGH_2_ is isomerized to PGE_2_ by a terminal PGE synthase (PGES) [6,7]. It has been shown in rats [8–14] and mice [15,16] that COX-2 and microsomal PGES-1 (mPGES-1) are transcriptionally up-regulated in endothelial and perivascular cells of brain microvessels between 1.5 and 12 h after administration of pyrogenic doses of bacterial lipopolysaccharide (LPS). Furthermore, Scammell et al. [17] have shown that microinjection of the COX inhibitor ketorolac into the preoptic region attenuates the febrile response over 1.5–6 h after intravenous (i.v.) injection of LPS in rats. These results indicate that febrigenic PGE_2_ is produced centrally.

It should be considered, however, that the initiation of fever precedes by approximately 1 h the earliest time point at which PGE_2_-synthesizing enzymes have been shown to be up-regulated in the brain. In a thermoneutral environment, i.v. LPS typically causes in rats and mice a polyphasic fever, and the first phase of this response starts at approximately 0.5 h post-LPS [18,19]. Because the first phase is sensitive to ambient temperature and can be readily masked by the stress hyperthermia associated with animal handling and LPS injection [19,20], this phase often escapes detection and remains the least studied component of the febrile response. The first phase of LPS fever was not investigated in any of the abovementioned studies of the source of febrigenic PGE_2_. We [21–23] and others [24–26] have hypothesized that, unlike the second and subsequent febrile phases, the first phase of fever is triggered by peripherally produced PGE_2_. Over the last two decades, several studies have attempted to test this hypothesis, but the results obtained have been inconclusive, contradictory, or incomplete (for details, see Results and Discussion). In particular, the location (inside or outside the brain) and phenotypes of the cells involved in the initiation of fever are unknown, as are the steps of the PGE_2_-synthesizing cascade that are initially activated to trigger the fever response. By closing these gaps, the present study identifies the cellular and molecular bases of the initiation of fever.

## Results/Discussion

The question as to whether peripherally (i.v. or intra-arterially) administered PGE_2_ causes fever remains controversial. Although there are reports of peripherally injected PGE_1_ and PGE_2_ being pyrogenic in several species of laboratory animals [24,27], there are at least as many documented failures to induce fever by peripheral administration of PGE [24,28,29]. The latter, negative results can be explained, at least partially, as due to self-aggregation of PGE in aqueous solutions and the subsequent loss of biological activity. Indeed, PGE_2_ was found to be highly pyrogenic in rabbits when infused in an albumin-bound (monomeric), but not in a free (aggregated) form [21]. Albumin is the principal carrier of PGE_2_ in the circulation, and up to 99% of circulating PGE_2_ is albumin-bound [30].

**Figure 1:**
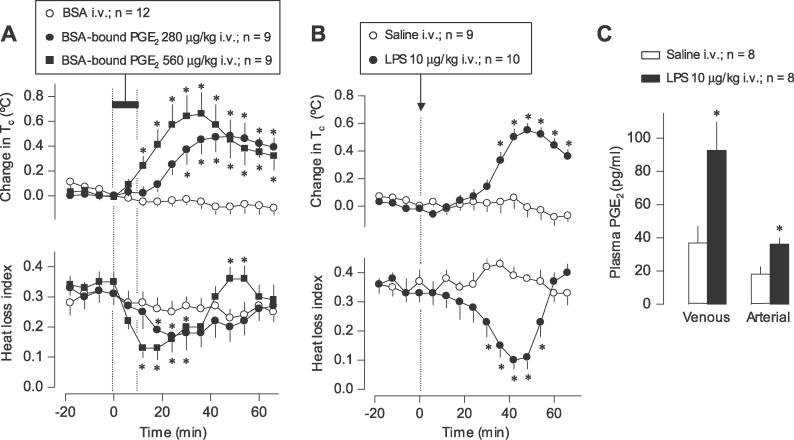
Circulating PGE_2_ Initiates LPS Fever in Rats: Circumstantial Evidence (A) The effects of i.v. infusion (horizontal bar) of BSA-bound PGE_2_ or BSA on T_c_ and heat loss index of rats kept at a neutral ambient temperature (30 °C). (B) The effects of i.v. bolus injection (arrow) of LPS or saline on the same parameters. Change in T_c_ was calculated by subtracting the T_c_ value at a given point from that at the start of infusion or injection (time zero). In (A), the absolute T_c_s at time zero were 38.3 ± 0.1 °C, 38.5 ± 0.1 °C, and 38.4 ± 0.2 °C for the groups treated with BSA and with the lower and higher doses of BSA-bound PGE_2_, respectively. In (B), initial T_c_s were 38.2 ± 0.1 °C and 38.3 ± 0.1 °C for the groups treated with saline and LPS, respectively. The heat loss index was calculated as a quotient of two temperature gradients: skin-ambient and colonic-ambient; this index varies between 0 (maximal vasoconstriction) and 1 (maximal vasodilation) [33]. (C) The levels of PGE_2_ in the venous and arterial blood of rats 40 min after i.v. injection of LPS or saline at thermoneutrality. This time point corresponds to the maximal thermoeffector activity (minimal heat loss index) to produce the first phase of LPS fever as shown in (B). All doses are indicated. Means ± SE are presented. The number of rats in each group *(n)* is indicated. An asterisk (*) indicates a significant difference from the BSA- or saline-treated group (*p* < 0.05; two-way analysis of variance for repeated measures followed by the Tukey test in [A] and [B]; Student *t*-test in [C]).

In the present study, a 2:1 (molar ratio) PGE_2_–albumin complex was prepared by adding PGE_2_ (all reagents are from Sigma-Aldrich, St. Louis, Missouri, United States, unless specified otherwise) and bovine serum albumin (BSA) to pyrogen-free saline, and then sonicating this mixture for 3 min and incubating it at 37 °C for 1 h. In a thermoneutral environment, the rats were infused i.v. with BSA-bound PGE_2_ (280 or 560 μg/kg, 100 μl/kg/min, 10 min). Based on the assumptions that PGE_2_ is evenly distributed in the extracellular compartment (20% of the body mass) and that its half-life is 1 min [31], it can be estimated that the protocol used elevates the plasma concentration of PGE_2_ by 350 pg/ml (low dose) or 700 pg/ml (high dose) at 12 min after the beginning of infusion. These concentrations are within the physiological range [24,32]. Whereas BSA had no thermoregulatory effect, the PGE_2_–BSA complex caused a dose-dependent rise in deep body (colonic) temperature (T_c_; Figure 1A). This fever response was brought about, at least in part, by tail skin vasoconstriction, as evident from a decrease in the heat loss index (the quotient of two temperature gradients: skin-ambient/colonic-ambient [33]). Hence, when administered in its most relevant form (albumin complex) and at physiologically relevant doses, peripheral PGE_2_ is pyrogenic in rats.

How circulating, albumin-bound PGE_2_ causes fever remains speculative. Activation of vagal afferents by PGE_2_ has been proposed [34], but the fact that vagotomy does not affect the first febrile phase (for discussion, see [35,36]) makes this mechanism unlikely. An alternative scenario seems more plausible. Binding to albumin prevents the rapid enzymatic inactivation of PGE_2_ [37,38], thus allowing it to reach a distant site. A good candidate for such a site is the preoptic hypothalamus, which is highly sensitive to the pyrogenic effect of PGE_2_ [39]. Once dissociated from albumin at the target site, PGE_2_ may be carried into the brain tissue by transporters expressed at the blood–brain barrier (BBB) [6,26,40]. It should be noted, however, that this scenario is speculative and needs to be tested experimentally.

Having shown that peripheral PGE_2_ is pyrogenic in rats, we asked whether blood levels of PGE_2_ are elevated at the onset of the first phase of LPS fever. Fever was induced by administering 0111:B4 Escherichia coli LPS (10 μg/kg) nonstressfully via the extension of a preimplanted venous (jugular) catheter to rats kept in a thermoneutral environment (see Materials and Methods for details). The first febrile phase started at approximately 30-min post-LPS and was brought about, at least partially, by tail skin vasoconstriction (Figure 1B). At 40 min (the time corresponding both to the maximal rate of rise in body temperature and to the maximal thermoeffector activity that underlies this rise), samples of venous and arterial blood were collected from LPS-treated (febrile) and saline-treated (afebrile) rats, and the concentration of PGE_2_ in the venous and arterial blood was measured by enzyme immunoassay. The venous blood gathers PGE_2_ synthesized in the tissues, and the arterial blood delivers it to the brain, the presumptive site of the febrigenic action of circulating PGE_2_ [39]. Consistent with the marked catabolism of PGE_2_ in the lungs [41], the level of PGE_2_ was lower in the arterial than in the venous blood plasma in both afebrile and febrile rats (Figure 1C). However, both the venous and arterial concentrations of PGE_2_ were substantially (~2.5 times) higher in the febrile rats as compared to the afebrile controls. These data show that the level of circulating PGE_2_, most importantly in the arterial blood, is increased at the onset of the first febrile phase.

**Figure 2:**
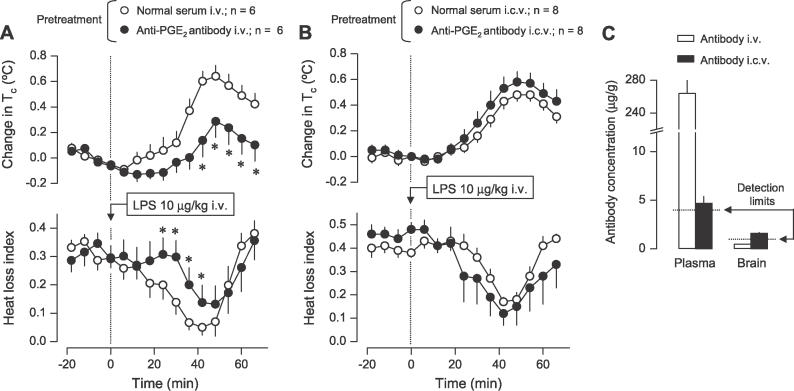
Circulating PGE_2_ Initiates LPS Fever in Rats: Direct Evidence (A) The effects of i.v. infusion (100 μl/kg/min, 120 min) of the anti-PGE_2_ antibody or normal serum 18 h before the experiment (pretreatment) on the T_c_ and heat loss index responses of rats injected (arrow) with LPS at thermoneutrality (30 °C). (B) The effects of the i.c.v. infusion (2.7 μl/min, 15 min) of the same anti-PGE_2_ antibody or normal serum 18 h before the experiment (pretreatment) on the same responses. Note that the i.c.v. infusion was aimed at testing whether minute amounts of the antibody in the brain are sufficient to suppress LPS fever (and not at testing whether fever is altered by neutralization of PGE_2_ in the brain). Change in T_c_ was calculated by subtracting the T_c_ value at a given point from that at the time of injection (time zero). In (A), the absolute T_c_s at time zero were 38.2 ± 0.1 °C and 38.1 ± 0.2 °C for the groups treated with i.v. normal serum and antibody, respectively. In (B), the initial T_c_s were 38.4 ± 0.1 °C and 38.2 ± 0.2 °C for the groups treated with i.c.v. normal serum and antibody, respectively. (C) The levels of anti-PGE_2_ antibody in the blood plasma and whole brain of rats pretreated with i.v. or i.c.v. antibody. Blood samples and brains were collected immediately after the temperature responses were recorded, i.e., approximately 20 h after pretreatment with the antibody. Antibody levels (means ± SE) are expressed as microgram of neat antibody per gram of either plasma or brain tissue. The detection limit for each assay and the number of rats in each group *(n)* are indicated. An asterisk (*) indicates a significant difference from the group pretreated with normal serum (*p* < 0.05; two-way analysis of variance for repeated measures followed by the Tukey test).

Several studies aimed at determining the source of febrigenic PGE_2_ have compared the antipyretic effects of nonsteroidal anti-inflammatory drugs administered peripherally (i.v. or intraperitoneally) and centrally (intracerebroventricularly [i.c.v.]). The drugs used included indomethacin [25], nimesulide [32], and keterolac (present study; unpublished data). All these studies faced multiple methodological problems, including acute thermoregulatory effects of the drug administered i.c.v. (present study), the ability of drugs to cross the BBB, and consequently, their tendency to be distributed evenly between the peripheral compartment and the brain [32]. We proposed [6] that selective neutralization of circulating PGE_2_ using an antibody is a better approach to test the hypothesis that peripherally produced PGE_2_ initiates fever. Being large proteins (160 kDa), antibodies cannot cross the BBB; this eliminates uncertainty common in experiments involving nonsteroidal anti-inflammatory drugs. The antibody used in the present study was raised against a PGE_2_–BSA complex in rabbits. It displayed a high affinity to PGE_2_ (association constant of 6.3 × 10^10^ M^−1^, as determined by Scatchard plot) and a low cross-reactivity with other prostanoids (<15% for PGF_1α_ and PGB_2_, and <9% for PGA_2_, PGF_2α_, and PGB_1_). The rats were pretreated i.v. with the anti-PGE_2_ antibody (neat antiserum; 100 μl/kg/min, 120 min) or with normal rabbit serum, and LPS was injected 18 h later, i.e., at the time when the injected antibody is expected to achieve a steady-state level in the circulation [42]. The results of this experiment are shown in Figure 2A–2C. The antibody (but not normal serum) suppressed the first phase of LPS fever: both the rise in T_c_ and the associated decrease in the heat loss index were delayed and significantly attenuated (Figure 2A). Immediately after the temperature response was recorded, a sample of venous blood and the whole brain (cleared of blood) were collected for immunoenzymatic determination of the anti-PGE_2_ antibody. The antibody was found at a high concentration in the blood plasma, but was below the detection limit in the brain tissue (Figure 2C). To rule out the possibility that a minute, undetectable amount of antibody in the brain might have accounted for the suppression of fever, we administered a low dose (2.7 μl/min, 15 min) of the anti-PGE_2_ antibody or normal serum i.c.v., and injected the rats with LPS 18 h later. The rats injected with the anti-PGE_2_ antibody i.c.v. had a detectable level of the antibody in the brain (Figure 2C), but their febrile response to LPS was unaffected (Figure 2B). A large fraction of the antibody given i.c.v. leaked into the blood, presumably reflecting the asymmetric nature of the BBB (its major role is to limit transport in the blood-to-brain direction, but not in the opposite direction) or possibly because the BBB was breached in this experimental group by the implanted i.c.v. cannula. Importantly, however, the plasma antibody concentration in the rats treated with the i.c.v. antibody was approximately 60 times lower than that in the rats treated with the i.v. antibody (Figure 2C). It is concluded that minute amounts of the anti-PGE_2_ antibody in the brain (even when detectable) are not sufficient to suppress the initiation of fever, and that the cause of the delayed and attenuated first febrile phase observed in the rats pretreated with i.v. antibody was neutralization of PGE_2_ outside the BBB.

**Figure 3:**
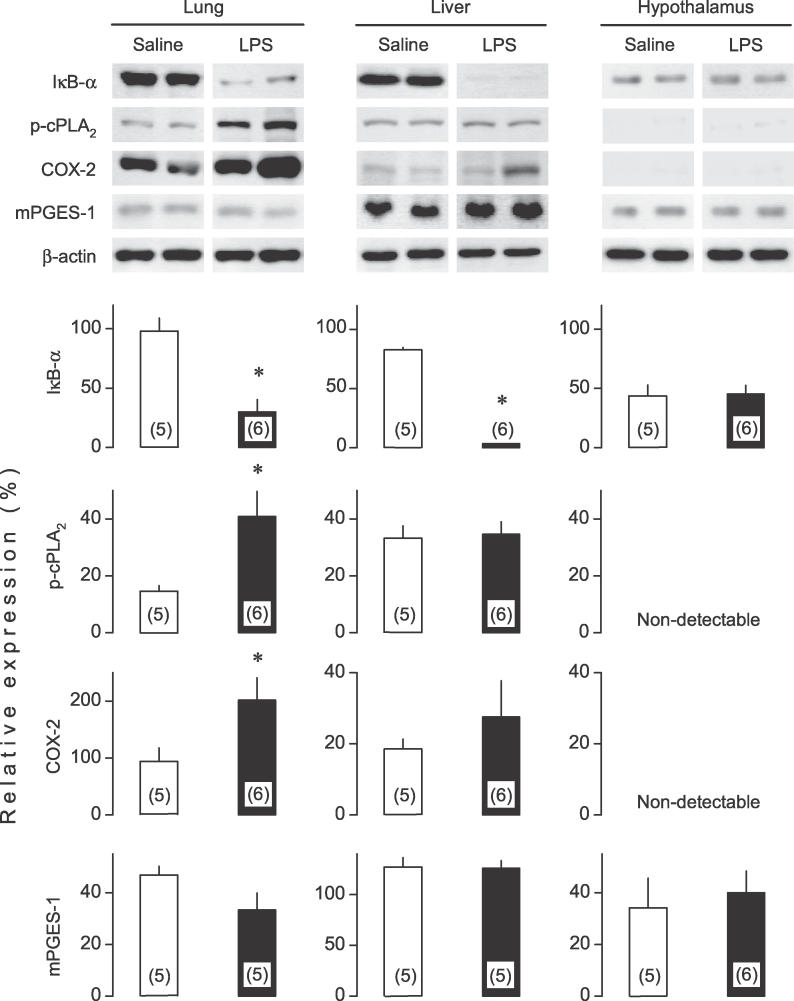
Mechanism of Activation of PGE_2_ Synthesis at the Onset of LPS Fever in Rats Tissue contents of the following proteins are shown: IκB-α (an inhibitor of nuclear factor-κB), three PGE_2_-synthesizing enzymes (p-cPLA_2_, COX-2, and mPGES-1), and β-actin (a “housekeeping” protein). These proteins were determined by Western blot in the lung, liver, and hypothalamus. The tissue samples were collected 40 min after i.v. injection of LPS (10 μg/kg) or saline at thermoneutrality. This time point corresponds to the maximal thermoeffector activity to produce the first phase of LPS fever (see Figure 1B). Electrophoretograms of two representative animals from each group are shown on top. The expression of each protein of interest (relative to the expression of β-actin) is shown on bottom (means ± SE); the number of rats *(n)* is shown in parenthesis. An asterisk (*) indicates a significant difference from the saline-treated group (*p* < 0.05; Student *t*-test).

**Figure 4:**
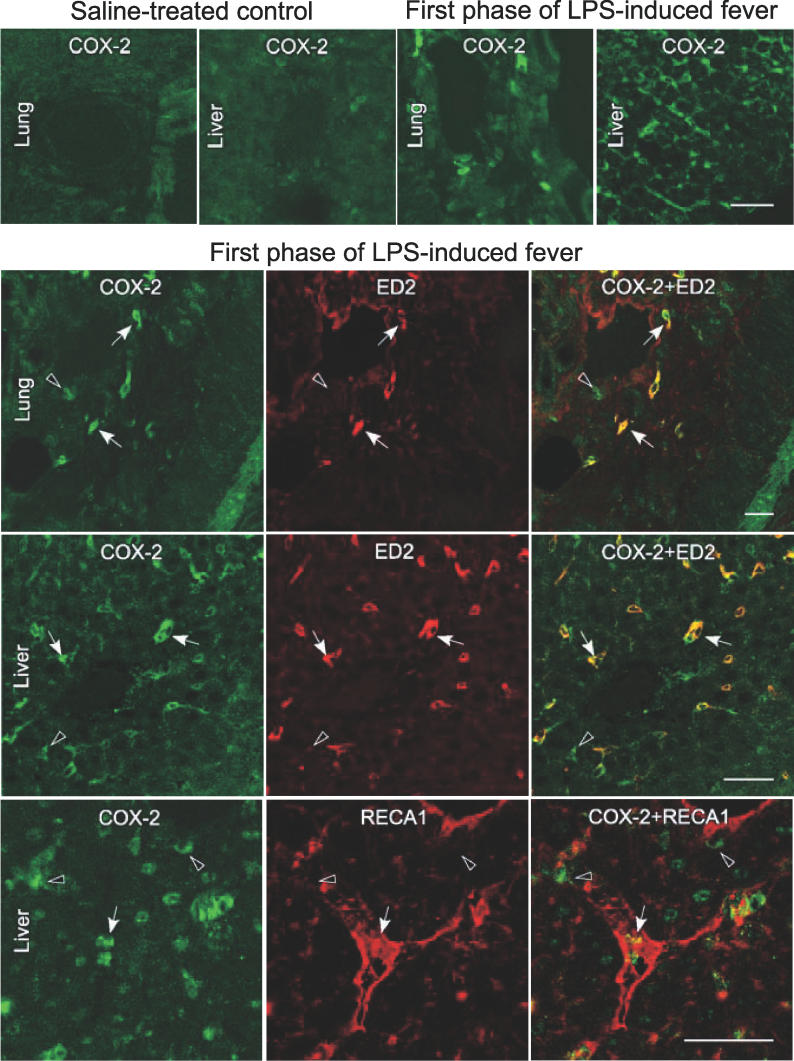
Identification of the Pulmonary and Hepatic Cells Producing PGE_2_ at the Onset of LPS Fever in Rats Immunolocalization of COX-2 in the lung and liver and identification of the cell types expressing this enzyme. Top row: tissue localization of COX-2 (green immunofluorescence) in the lung and liver of rats at 40 min after i.v. injection of saline or at the onset of the first febrile phase (i.e., 40 min after i.v. injection of LPS, 10 μg/kg) at thermoneutrality. Next rows: dual localization of LPS-induced COX-2–immunoreactivity (green; left column) with either the macrophage marker ED2 or the endothelial cell marker RECA1 (red; middle column) in the lung and liver at the onset of the first febrile phase. Doubly labeled cells appear yellow in the merged confocal images (right column). White arrows and black arrowheads mark examples of doubly and singly (COX-2 only) labeled cells, respectively. Scale bars represent 40 μm.

Having demonstrated that circulating PGE_2_ is indeed responsible, at least partially, for triggering LPS-induced fever, we investigated which step of the PGE_2_ biosynthetic pathway is activated at the onset of the febrile response. Previously, we reported that the onset of the first phase of LPS fever is associated with large increases of COX-2 and mPGES-1 mRNAs in the lung and liver and with a moderate increase of COX-2 (but not mPGES-1) mRNA in the hypothalamus [22]. However, it remained to be determined whether the observed transcriptional changes translate into changes in the corresponding protein contents at such an early time point (40 min) after LPS administration. We had also shown [22] that neither cytosolic PLA_2_-α (cPLA_2_-α) nor either of the two secretory PLA_2_ studied (II and V) is transcriptionally up-regulated at the onset of fever. This finding, however, does not exclude the possibility that cPLA_2_ is activated posttranscriptionally by phosphorylation, the principal mechanism of activation for this enzyme [43]. In the present study, we determined the contents of phosphorylated cPLA_2_ (p-cPLA_2_), COX-2, and mPGES-1 proteins by Western blot in the lung, liver, and hypothalamus at 40 min after injection of LPS or saline, a time that corresponds to the onset of the first febrile phase in LPS-treated rats (Figure 1B). COX-2–positive cells were also studied in all three tissues by immunohistochemistry using two different protocols of sample preparation (see Material and Methods). None of the enzymes studied was increased at the protein level in the hypothalamus of the LPS-treated rats as compared to the saline-treated controls (Figure 3). Neither did the immunohistochemical analysis reveal any increase in the number of COX-2–positive hypothalamic cells at the onset of fever, although the same antibody readily detected a surge in the number of COX-2–positive endotheliocytes in the hypothalamic microvasculature at later stages of LPS fever, in both the present study (positive controls; unpublished data) and previous studies [11,12]. In the lung, LPS increased the contents of p-cPLA_2_ and COX-2 (Figure 3), and augmented the number of cells containing COX-2 (Figure 4), but did not alter the protein level of constitutively expressed mPGES-1 (Figure 3). In the liver, the immunohistochemical analysis (which is more sensitive) revealed a surge in the number of COX-2–positive cells at the onset of fever (Figure 4), whereas the Western blot analysis (less sensitive) found a tendency for an increase in the overall content of COX-2 and no changes in the content of either p-cPLA_2_ or mPGES-1 (Figure 3). We also found that inflammatory signaling (assessed by a decrease in the content of the nuclear factor-κB inhibitor, IκB-α [44]) was activated in the lung and liver, but not in the hypothalamus, at the onset of LPS fever (Figure 3).

These results show that the onset of the first febrile phase is associated with activation of inflammatory signaling and increased PGE_2_ synthesis in the periphery. The early activation of PGE_2_ synthesis involves phosphorylation of cPLA_2_ (lung) and transcriptional up-regulation of COX-2 (lung and liver). Transcriptional up-regulation is the main (although not the only [45,46]) mechanism of activation for this enzyme [6,7]. Hence, the increased circulating level of PGE_2_ at the onset of the first febrile phase may be explained by the following enzymatic events in the lung and liver: production of arachidonic acid by activated (phosphorylated) cPLA_2_ → conversion of arachidonic acid to PGH_2_ by up-regulated COX-2 → isomerization of PGH_2_ into PGE_2_ by constitutively expressed mPGES-1. Whereas the physiological importance of cPLA_2_ and mPGES-1 in the first febrile phase remains to be confirmed in studies with pharmacological or genetic blockade of these enzymes, the indispensable role of COX-2 (and the uninvolvement of COX-1) in the first phase of LPS fever have been demonstrated in our recent study in knockout mice [47].

Preferential location of the synthesis of febrigenic PGE_2_ in the liver and lungs (but not in the brain) deserves special discussion. The fact that the i.v. antibody attenuated the first febrile phase but did not abolish it completely (Figure 2A) may be due to incomplete neutralization of circulating PGE_2_. However, it may also reflect a contribution of centrally produced PGE_2_ (e.g., by a small number of hypothalamic cells that express COX-2 constitutively) to the development of the first phase of LPS fever. Although we cannot rule out such a contribution, it is noteworthy that multiple methods used in our present and previous [22] studies (Table 1) found a profound activation of PGE_2_ synthesis in the periphery, but hardly any signs (none at the protein level) of activation of hypothalamic PGE_2_ synthesis.

**Table 1:**
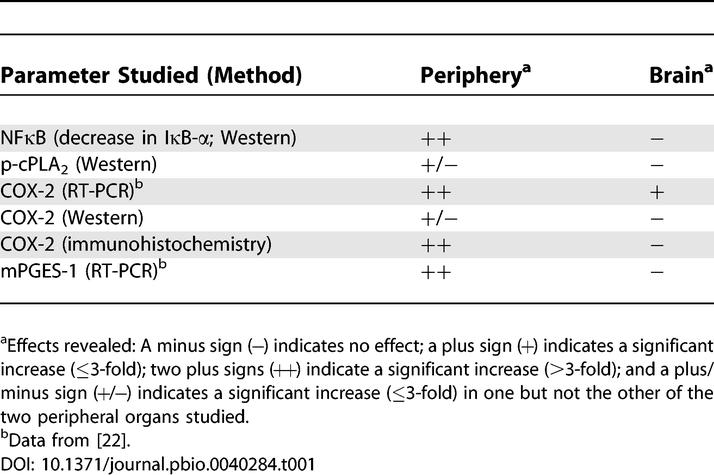
Inflammatory Signaling and PGE_2_ Synthesis Are Selectively Activated at the Onset of the First Phase of LPS Fever in the Periphery (Lung and Liver) but Not in the Brain (Hypothalamus)

To identify the pulmonary and hepatic producers of PGE_2_, we first determined how the cells that become COX-2 positive at the onset of LPS fever relate to the histological elements revealed by eosin staining; this analysis was performed in freshly frozen samples. In the lung, COX-2–positive cells were found to cluster around alveoli, often forming what looked like cell chains (unpublished data). In the liver, the parenchyma did not stain for COX-2, and the vast majority of COX-2–positive cells were located in the stromal compartment, often in close proximity to sinusoids. Some COX-2–positive cells were also found around the central vein (a small vein that gathers the blood from sinusoids) and in the visceral peritoneum covering the liver (unpublished data). We then double-stained lung and liver for COX-2 and either the macrophage marker ED2 [48] or the endothelial marker RECA1 [49]; this analysis was performed in paraformaldehyde-fixed samples (Figure 4). In the lung, 89 ± 6% (mean ± standard error [SE] of five samples) of COX-2–positive cells were macrophages (ED2 positive), and 11% were unidentified (ED2 and RECA1 negative). In the liver, 83 ± 2% of the COX-2–positive cells were macrophages (ED2 positive), 9 ± 1% were endotheliocytes (RECA1 positive), and 8% remained unidentified. The key role of macrophages in the initiation of fever agrees with our recent finding that the first febrile phase depends entirely on the recognition of LPS (via the Toll-like receptor-4) by bone marrow-derived cells [50].

In summary, the present study shows that the first phase of LPS fever is initiated (at least partially) by PGE_2_ that originated in peripheral tissues. Activation of PGE_2_ synthesis at the onset of the first phase of LPS fever involves phosphorylation of cPLA_2_, transcriptional up-regulation of COX-2, and possibly other mechanisms. The vast majority of the PGE_2_-producing cells are macrophages. These findings challenge the predominant view that fever is initiated exclusively by inflammatory mediators produced at the level of the BBB. These findings, however, do not contradict the principal role of the centrally produced PGE_2_ in the second and subsequent febrile phases.

## Materials and Methods

### Animals.

The study was conducted in male Long-Evans rats weighing 300–400 g (Charles River, Wilmington, Massachusetts, United States). The rats were habituated (seven daily training sessions, 4 h each) to spending time in artificial “rat holes,” cylindrical confiners made of stainless steel wire [20,22,23]. The same confiners were used later in the experiments. Each rat was used in only one experiment. The protocols were approved by the St. Joseph's Hospital Animal Care and Use Committee.

### Surgery and instrumentation.

Under ketamine-xylazine-acepromazine anesthesia (55.6, 5.5, and 1.1 mg/kg, respectively, intraperitoneally) and antibiotic (enrofloxacin, 1.1 mg/kg, subcutaneously) protection, each rat was subjected to chronic catheterization of the jugular vein as described elsewhere [22]. The catheters were flushed with heparinized (10 U/ml) saline on Days 1 and 3 postsurgery. The experiments were performed on Day 5. On the day of the experiment, each rat was placed in a wire confiner and equipped with two copper-constantan thermocouples: one for recording T_c_ and the other for recording tail skin temperature. The colonic thermocouple was inserted 10 cm beyond the anal sphincter and fixed to the base of the tail with adhesive tape. The skin thermocouple was positioned at the boundary of the proximal and middle thirds of the tail, on its lateral surface, and was insulated from the environment with tape. The thermocouples were plugged in to a data logger (Cole-Parmer, Vernon Hills, Illinois, United States), which was connected to a personal computer. The rats in their confiners were placed in a climatic chamber (Forma Scientific, Marietta, Ohio, United States) set to a neutral ambient temperature of 30.0 °C. Their jugular catheters were extended with lengths of PE-50 tubing filled with saline, thus permitting i.v. drug administration to be performed in a stress-free fashion, from outside the chamber.

### Tissue harvesting.

Immediately before collection of blood and tissue specimens, the rats were anesthetized with ketamine-xylazine-acepromazine (5.56, 0.55, and 0.11 mg/kg, respectively, i.v.). Blood samples were collected either from the inferior vena cava (venous blood) or left ventricle (arterial blood). Each sample was transferred to an eppendorf tube containing EDTA and indomethacin (final concentrations: 1 mg/ml and 10 μM, respectively). The collected blood was immediately centrifuged (3,000 *g,* 10 min, 4 °C), and the resulting plasma was stored at −80 °C.

For collection of tissue samples for the immunoassay and Western blot protocols, each rat was perfused through the left ventricle (right atrium cut) with 100 ml of 10 mM phosphate-buffered saline (PBS; pH 7.4). The right medial lobe of the liver, the medial lobe of the right lung, and either the hypothalamus (for Western blot) or the entire brain were collected rapidly and snap frozen in liquid nitrogen. The samples were stored at −80 °C.

Tissue specimens for the immunohistochemistry protocols were collected using two methods. For immunohistochemical analysis of freshly frozen (non-fixed) tissues, a rat was perfused with PBS (50 ml). The liver, right lung, and brain were excised, frozen in dry ice powder, and stored at −80 °C. The specimens were cryosectioned (section thickness, 14 μm) immediately before the analysis. For immunohistochemical analysis of fixed tissues, a rat was perfused with 50 ml of heparinized (10 U/ml) PBS followed by 200 ml of a 4% paraformaldehyde solution in PBS. The liver, right lung, and brain were excised, postfixed in the paraformaldehyde solution for 2 h, transferred to PBS containing 20% sucrose, and kept in this solution at 4 °C for 24 h. The specimens were then frozen, cryosectioned (section thickness, 30 μm), and immediately subjected to the immunohistochemical analysis.

### Immunoassays.

PGE_2_ was measured in plasma by enzyme immunoassay using a commercially available kit (Cayman, Ann Arbor, Michigan, United States); the samples were prepared according to the manufacturer's instructions. The levels of anti-PGE_2_ antibody in the plasma and brain were assayed by enzyme immunoassay involving a conjugate of four proteins: goat anti-mouse IgG (which coated all wells of a 96-well plate), mouse anti-rabbit IgG, rabbit anti-PGE_2_ IgG (sample or standard), and PGE_2_-horseradish peroxidase complex (Amersham, Piscataway, New Jersey, United States). The conjugate was developed by adding the peroxidase substrate 3,3′,5,5′-tetramethylbenzidine; the reaction product had maximal absorbance at 630 nm. Samples of blood plasma were diluted (1:256) in 100 mM Tris-buffered saline (pH 7.4) before the assay. Each brain was homogenized in 4 ml of a 100 mM NaOH solution containing 0.2% sodium dodecyl sulfate. After the homogenate was cleared by centrifugation (14,000 *g,* 10 min, 6 °C), the supernatant was neutralized with 100 mM HCl and diluted (1:10) with Tris-buffered saline. The detection limits for blood plasma (4 μg/g) and brain tissue (1 μg/g) were determined as the triple noise-level concentrations.

### Western blot.

The Western blot protocol was identical to that used to measure IκB-α and β-actin in our earlier study [51], except that here we also measured p-cPLA_2_, COX-2, and mPGES-1. The following primary rabbit polyclonal antibodies were used (dilutions indicated): anti-IκB-α (1:1,000; Cell Signaling, Beverly, Massachusetts, United States), anti-β-actin (1:1,000), anti–p-cPLA_2_ (1:500; Cell Signaling), anti–COX-2 (1:1,000; Cayman), and anti-mPGES-1 (1:1,000; Cayman). The primary antibodies revealed immunoblot bands corresponding to the following molecular masses: 36 kDa for IκB-α; 42 kDa for β-actin; 105 kDa for p-cPLA_2_; 72 kDa for COX-2; and 16 kDa for mPGES-1.

### Immunohistochemistry.

Single immunofluorescence was used to visualize and localize COX-2–positive cells in freshly frozen [11,12] or paraformaldehyde-fixed [52] samples of lung, liver, and hypothalamus. Dual immunofluorescence protocols were used to verify co-localization of COX-2 with ED2 (a macrophage marker) or RECA1 (an endotheliocyte marker) in paraformaldehyde-fixed samples of lung and liver [52]. The fraction of COX-2–immunoreactive cells that were also immunoreactive to ED2 or RECA1 was determined in serial (10–15) sections (30 μm-thick) collected at 150-μm intervals through the right lobe of the liver or right lung. Images of merged confocal channels were collected from 104-μm^2^ fields (nine for each section).

## Abbreviations

BBB: blood–brain barrier
BSA: bovine serum albumin
COX: cyclooxygenase
cPLA_2_: cytosolic phospholipase A_2_
i.c.v.: intracerebroventricular(ly)
i.v.: intravenous(ly)
LPS: lipopolysaccharide
mPGES: microsomal prostaglandin E synthase
PBS: phosphate-buffered saline
p-cPLA_2_: phosphorylated cytosolic phospholipase A_2_
PG: prostaglandin
PLA_2_: phospholipase A2
SE: standard error, T_c_, colonic temperature
